# Genotoxic and Antigenotoxic Assessment of Chios Mastic Oil by the *In Vitro* Micronucleus Test on Human Lymphocytes and the *In Vivo* Wing Somatic Test on *Drosophila*

**DOI:** 10.1371/journal.pone.0130498

**Published:** 2015-06-25

**Authors:** Dimitris Vlastos, Elena Drosopoulou, Ioanna Efthimiou, Maximos Gavriilidis, Dimitra Panagaki, Krystalenia Mpatziou, Paraskevi Kalamara, Despoina Mademtzoglou, Penelope Mavragani-Tsipidou

**Affiliations:** 1 Department of Environmental and Natural Resources Management, University of Patras, Agrinio, Greece; 2 Department of Genetics, Development and Molecular Biology, School of Biology, Faculty of Science, Aristotle University of Thessaloniki, Thessaloniki, Greece; University of Hawaii Cancer Center, UNITED STATES

## Abstract

Chios mastic oil (CMO), the essential oil derived from *Pistacia lentiscus* (L.) var. *chia* (Duham), has generated considerable interest because of its antimicrobial, anticancer, antioxidant and other beneficial properties. In the present study, the potential genotoxic activity of CMO as well as its antigenotoxic properties against the mutagenic agent mitomycin-C (MMC) were evaluated by employing the *in vitro* Cytokinesis Block MicroNucleus (CBMN) assay and the *in vivo* Somatic Mutation And Recombination Test (SMART). In the *in vitro* experiments, lymphocytes were treated with 0.01, 0.05 and 0.10% (v/v) of CMO with or without 0.05 *μ*g/ml MMC, while in the *in vivo* assay *Drosophila* larvae were fed with 0.05, 0.10, 0.50 and 1.00% (v/v) of CMO with or without 2.50 *μ*g/ml MMC. CMO did not significantly increase the frequency of micronuclei (MN) or total wing spots, indicating lack of mutagenic or recombinogenic activity. However, the *in vitro* analysis suggested cytotoxic activity of CMO. The simultaneous administration of MMC with CMO did not alter considerably the frequencies of MMC-induced MN and wing spots showing that CMO doesn’t exert antigenotoxic or antirecombinogenic action. Therefore, CMO could be considered as a safe product in terms of genotoxic potential. Even though it could not afford any protection against DNA damage, at least under our experimental conditions, its cytotoxic potential could be of interest.

## Introduction

Natural products have been proven to possess multiple biological properties and gained significant interest for the development of various human-related applications, including medical treatments. While most studies are focused on isolated compounds, there is increasing evidence that natural combinations of phytochemicals in extracts show enhanced properties [1,2].

Chios mastic gum, the resin of the endemic bush *Pistacia Lentiscus* (L.) var. *chia* (Duham) from the Greek island Chios [3,4], has received much attention in recent years. Both the resin itself and its essential oil, Chios mastic oil (CMO), have been thoroughly studied for their antibacterial, antimicrobial, anti-inflammatory and antioxidant activity [5–9] and they have shown great potential as anticancer and cytotoxic agents [10]. CMO is extracted from the Chios mastic resin through steam distillation. Containing a large variety of therapeutic, aromatic and flavoring ingredients, it is used in the food industry as well as in health and care products [11]. Its major compounds are α-pinene and β-myrcene, constisting more than 85% of the total concentration, while many other minor constituents have also been identified by GC-MS analysis and FT-Raman spectroscopy [5,6,12].

Emboldened by our previous findings for antigenotoxicity and lack of genotoxicity of another mastic product, Chios mastic water (CMW) [1], in the present study we evaluated the possible cytotoxic, genotoxic and antigenotoxic activity of CMO with the cytokinesis block micronucleus (CBMN) assay and the somatic mutation and recombination test (SMART).

CBMN is a simple, rapid and sensitive *in vitro* assay for the detection of micronuclei (MN) in the cytoplasm of interphase human lymphocytes [13]. The formation of MN may be due to the inability of acentric chromosome fragments or whole chromosomes to migrate to the poles during the anaphase stage of cell. Therefore, it is possible through this assay to detect both aneugenic and clastogenic effects in cells that have undergone cell division after exposure to the test chemical [13,14].

SMART test in *Drosophila melanogaster* is a sensitive, low-cost and quick eukaryotic *in vivo* assay that enables the detection of a wide spectrum of genetic end points, including point mutations, deletions, chromosome aberrations, mitotic recombination and gene conversion [15,16]. The fruit fly, *D*. *melanogaster*, has been intensively used as a genetic model system for mutation research and genetic toxicology because of advantages, such as the extensive knowledge of its genetics, the ease of its laboratory maintenance and genetic manipulations and the high homology between fly and human genes [17–20].

Genotoxic and recombinogenic events as well as MN formation are reported to be associated with carcinogenesis [21,22]. In this context, our results are expected to contribute on the safety status of CMO, a commercially available product with a wide spectrum of biological activities and potential applications [5,6,23–25].

## Materials and Methods

### Chemicals

CMO was supplied by Chios Mastiha Growers’ Association (CMGA, Chios, Greece). Mitomycin C (MMC) and cytochalasin-B (Cyt-B) were purchased from Sigma (St. Louis, MO, USA). Ham’s F-10 medium, foetal bovine serum and phytohaemaglutinin were commercially supplied (Gibco, UK). Faure’s solution was prepared by mixing 100 g distilled H_2_O, 100 g chloral hydrate (C_2_H_3_Cl_3_O_2_), 40 g glycerine (C_3_H_8_O_3_) and 60 g arabic gum. All other chemicals and solvents were of the highest grade commercially available. Stocks of the compounds and solutions were stored at 4°C until use.

### Ethics statement

The study was approved by the Ethical Committee of the University of Patras. After written informed consent, two healthy nonsmoking male individuals (less than 30 years) were used as blood donors to establish whole blood lymphocyte cultures. According to the donors' declaration, they were not exposed to radiation, drug treatment or any viral infection in the recent past.

### CBMN assay in human lymphocytes *in vitro*

Blood samples were kept under sterile conditions in heparinized tubes. Whole blood (0.5 ml), 6.5 ml Ham’s F-10 medium, 1.5 ml foetal bovine serum and 0.3 ml phytohaemaglutinin to stimulate cell division, were added to the culture.

Dilution of CMO in ethanol (1:1 v/v) was conducted and it was subsequently added to final concentrations of 0.01, 0.05 and 0.10% (v/v) in culture volume either alone or in combination with 0.05 *μ*g/ml of MMC. The MMC concentration used in the present study has been previously used as positive control in the particular assay and cell type [1,26]. The CMO concentrations were selected based on a previous work by our group [1] proving the protective effect of CMW-aqueous extract of mastic resin which contains CMO at 0.5–1% (v/v) concentration [data from CMGA]- against the MMC-induced genotoxicity in the CBMN assay. Two identical sets of the experiment were conducted for all aforementioned concentrations as well as for positive and negative controls.

24 h after culture initiation the appropriate volumes of chemicals were added and 20 h later Cyt-B was added at final concentration of 6 *μ*g/ml in every culture. According to the scientific literature this concentration of Cyt-B has been proven best in the acquisition of a higher percentage of binucleated (BN) cells and a lower baseline MN frequency [27]. The incubation of cultures took place in a humidified atmosphere of 5% CO_2_ at 37°C. 72 h after the initiation of culture, cells were harvested and collected by centrifugation. 3:1 solution of Ham’s medium and milli-q H_2_O was used as a mild hypotonic treatment and cells were left for 3 min at room temperature. 10 min fixation (for at least 3 times) was then performed using a fresh 5:1 solution of methanol/acetic acid and finally cells were stained with 7% Giemsa [28–30].

A total of 2000 BN cells with preserved cytoplasm was scored per experimental point. Scoring of MN was conducted according to standard criteria [31,32] and performed manually by two, independently working, experienced researchers. The cytokinesis block proliferation index (CBPI) was applied so as to determine potential cytotoxicity and it was calculated by counting at least 1000 cells for each experimental point (500 cells per culture per donor). CBPI is given by the equation: CBPI = [M_1_ + 2M_2_ + 3(M_3_ + M_4_)]/N, where M_1_, M_2_, M_3_ and M_4_ correspond to the numbers of cells with one, two, three, and four nuclei and N is the total number of cells [33].

### SMART test in *Drosophila melanogaster in vivo*

Two *D*. *melanogaster* strains, the multiple wing hair strain (*mwh*, 3–0.3) with genetic constitution *fs(1)K10 w/Y;mwh se e/mwh se e* and the flare strain (*flr* ^*3*^, 3–38.8) with genetic constitution *y wco*/*y wco; flr3 se*/*TM2 Ubx130 se e* [34,35], were used in the present study. Description of the genetic markers is given in Lindsley and Zimm [34]. Insects were maintained at 24±1°C, at a photoperiod 16:8 (light:dark) on a yeast–glucose medium. The experiments were carried out as described in Vlastos et al. [1] following the principles and the basic procedures presented by Graf et al. [15,16]. Thus, eggs obtained by parental crosses between *flr* ^*3*^ virgin females and *mwh* males were collected during a six-hour period and 72±3 h later, the larvae were washed out of the bottles with Ringer’s solution and collected in a stainless steel strainer. Series of 40 larvae were transferred for chronic feeding to treatment vials containing 0.85 g of *Drosophila* Instant Medium (Carolina Biological Supply, Burlington, NC, USA) rehydrated with 4 ml of 0.05, 0.10, 0.50 and 1.00% (v/v) CMO alone or in combination with MMC. The above concentrations were used based on previous studies [10] as well as on a previous work of our group [1], where the aqueous extract of mastic resin, CMW, which contains CMO at 0.5–1% (v/v) concentration [data from CMGA], was found to have a protective role against the MMC-induced genotoxicity. MMC was used at final concentration of 2.50 μg/ml, which has previously been shown to be mutagenic in our system [1] and, thus, it also served as positive control. Larvae were fed on these culture media for the rest of their larval life (approximately 48 h). The trans-heterozygous (*mwh flr*+/ *mwh*+ *flr* ^*3*^) female flies that emerged from the cross mentioned above were selected and stored in 70% v/v ethanol-glycerol (1:1 v/v). Their wings were mounted in Faure’s solution and scored at 400x magnification for the presence of mosaic spots [15,34,36]. On the basis of the size, number, and type of cells showing malformed wing hairs, the spots were grouped into four categories: (i) small single spots (with one or two affected cells, either *mwh* or *flr* ^*3*^), (ii) large single spots (with three or more affected cells, either *mwh* or *flr* ^*3*^), (iii) twin spots (consisting of both *mwh* and *flr* ^*3*^ subclones), and (iv) total spots [15,34]. Single spots (*mwh* or *flr* ^*3*^) are produced by various genetic events including somatic point mutations, deletions and other types of structural rearrangements as well as by mitotic recombination between the two marker genes, while twin spots (*mwh* and *flr* ^*3*^) are produced exclusively by mitotic recombination occurring between the proximal marker *flr* ^*3*^ and the chromosome 3 centromere [34]. For comparative analysis, parallel experiments using either distilled water or ethanol solution (1%) were carried out as the negative controls. Ten replicates per treatment were performed. Since no considerable difference in survival rates of hatched flies from independent experiments was observed, approximately 50 wing samples per treatment were randomly selected for genotoxic analysis. All experiments were performed at 24±1°C and 60% RH. A total of about 600 wings were scored in this study.

### Statistical analysis

All results of the CBMN assay are expressed as the mean frequency ± standard error (MF ± se). The G-test for independence on 2x2 tables was used to perform the statistical analysis of the MN data. The chi-square test (*χ2* test) was used for the analysis of CBPI among each treatment. Differences at p < 0.05 were considered significant. The statistical softwares used for data analysis were the Origin 7.0 (OriginLab Corporation, Northampton, MA, USA), the Minitab statistical software (Minitab Inc., PA, USA), and the Statistical Package for Social Sciences (SPSS) for Windows, version 17.0.

Statistical analysis of the data derived by the SMART assay was done using the multiple-decision procedure [37,38] which is based on the conditional binomial test and the chi-squared test (K. Pearson’s criterion) [39,40]. A significance level of 5% was used. For the statistical assessment of antigenotoxicity, the frequencies of each type of spots per fly were compared in pairs (MMC versus MMC+CMO), using the nonparametric Mann-Whitney U-test [41], which was performed with SPSS.

## Results

### CBMN assay in human lymphocytes *in vitro*

CMO was studied for genotoxicity at three different concentrations, i.e. 0.01, 0.05 and 0.10% of total volume of human lymphocytes culture, and the results are summarized in Table 1.

**Table 1:** Frequencies of micronucleated binucleated cells (BNMN) and micronuclei (MN) as well as cytokinesis block proliferation index (CBPI) values in cultured human lymphocytes which have been treated with Chios mastic oil (CMO), mitomycin-C (MMC) (0.05 *μ*g/ml) and their mixture.

Treatment	BNMN MF (‰)±se	MN MF (‰)±se	CBPI MF (‰)±se
**Control**	2.5±0.5	2.5±0.5	1.95±0.09
**0.01% (v/v) CMO**	3.0±1.0	3.0±1.0	1.92±0.04
**0.05% (v/v) CMO**	5.0±1.0	5.0±1.0	1.65±0.03^1^
**0.10% (v/v) CMO**	2.5±0.5	2.5±0.5	1.41±0.02^1^
**MMC (0.05*μ*g/ml)**	56.0±3.0^1^	56.5±3.5^1^	1.71 ±0.00^1^
**0.01% (v/v) CMO + MMC (0.05*μ*g/ml)**	48.5±8.5^1^	49.0±8.0^1^	1.68 ±0.01^1^
**0.05% (v/v) CMO + MMC (0.05*μ*g/ml)**	56.0±1.0^1^	56.5±1.5^1^	1.48±0.02^1^ ^,^ ^a^
**0.10% (v/v) CMO + MMC (0.05*μ*g/ml)**	54.5±1.5^1^	56.5±0.5^1^	1.25±0.06^1^ ^,^ ^a^

BN: binucleated cells; BNMN: micronucleated binucleated cells; MN: micronuclei; CBPI: Cytokinesis Block Proliferation Index; CMO: Chios Mastic Oil; MMC: Mitomycin-C; MF (‰)±se, mean frequencies (‰)±standard error; MN were scored in 2000 binucleated lymphocytes per experimental point;

^1^ Significant difference compared to control at p<0.001;

^a^ Significant difference compared to MMC at p<0.001; G-test for BNMN and MN; *χ* ^*2*^ for CBPI

No statistically significant differences in the binucleated cells with micronuclei (BNMN) as well as in the MN frequencies were observed between control and CMO-treated cultures. The same CMO doses were also tested in combination with 0.05 *μ*g/ml MMC in order to identify whether and at which percentage CMO decreases its genotoxic effect. As expected, MMC alone provoked a statistically significant increase in MN (average 56.5 vs 2.5) and BNMN frequency (average 56.0 vs 2.5) as compared to control. This increase was maintained or slightly decreased after co-treatment with CMO and MMC (average 49.0–56.5 and 48.5–56.0 for MN and BNMN, respectively, for the different CMO concentrations). Thus, CMO did not counteract in a statistically significant way the genotoxic effects of MMC.

CMO was further tested for cytotoxicity with and without MMC by the determination of the CBPI index (Table 1). CMO decreased this index as compared to control (1.41–1.92 vs 1.95), with the difference reaching statistical significance at the two highest CMO doses. When CMO and MMC were co-administered to the lymphocytes culture, the addition of CMO reduced the CBPI observed by MMC alone (1.25–1.68 vs 1.71). This decline was statistically significant for the two highest CMO concentrations.

The size ratio of MN in the *in vitro* CBMN assay is an alerting index as effective as the fluorescence *in situ* hybridization (FISH) analysis for the discrimination of clastogenic and aneugenic effects [29,42]. Compared to the positive control size ratio of MN, no statistically significant decrease in small and large MN frequency in mixtures of CMO and MMC was observed (data not shown).

To summarize, CMO was not genotoxic at any of the applied concentrations, while it exerted cytotoxic activity at the highest concentrations. CMO did not statistically significantly reduce the genotoxic effect of MMC. On the other hand, the combination of CMO and MMC induced a significant decline of the CPBI index.

### SMART assay in *D*. *melanogaster in vivo*

CMO was examined for its possible mutagenic and recombinogenic activities *in vivo* at four concentrations [0.05, 0.10, 0.50 and 1.00% (v/v)] by the SMART assay. The evaluation of the antigenotoxic effect of CMO against the genotoxic damage induced by MMC was accomplished by co-treatment of the above doses of CMO with MMC at final concentration of 2.50 μg/ml. The results together with the negative control experiment are summarized in Table 2. No significant differences in the frequency of the observed spontaneous spots were found between the two negative controls (water control and 1% ethanol). Thus, for the statistical analysis the average spontaneous frequency of total spots (0.63) of the controls was used. The comparative screening for spontaneous and induced mutagenesis after chronic treatment of *Drosophila* larvae with CMO at the lowest concentrations (0.05 and 0.10% v/v) showed no significant differences (p >0.05) in the frequency of any type of spots in the treated and the negative control series, indicating absence of genotoxicity (Table 2). At the highest concentrations of CMO (0.50 and 1.00% v/v), even though a higher percentage of small spots was observed, the analysis of the total spots gave inconclusive result (Table 2). However, this result could be interpreted as having minor biological significance, since the frequency of total mutant clones was not highly different from the control (0.82–0.88 vs 0.63).

**Table 2:** Frequencies of small, large, twin and total mosaic spots in *D*.*melanogaster* wings of individuals treated with Chios mastic oil (CMO), mitomycin-C (MMC) (2.5 *μ*g/ml) or their mixture.

Treatment	Number of wings	Frequency of spots per wing and diagnosis^1^			
		Small single spots	Large single spots	Twin spots	Total spots
**Control**	48	0.46 (22)	0.13 (6)	0.04 (2)	0.63 (30)
**0.05% (v/v) CMO**	48	0.42 (20) -	0.17 (8) -	0.04 (2) i	0.63 (30) -
**0.10% (v/v) CMO**	49	0.39 (19) -	0.10 (5) -	0.12 (6) i	0.61 (30) -
**0.50% (v/v) CMO**	51	0.71 (36) i	0.08 (4) -	0.10 (5) i	0.88 (45) i
**1.00% (v/v) CMO**	50	0.74 (37) +	0.04 (2) -	0.04 (2) i	0.82 (41) i
**MMC (2.5*μ*g/ml)**	50	1.38 (69) +	0.44 (22) +	0.46 (23) +	2.28 (114) +
**0.05% (v/v) CMO + MMC (2.5*μ*g/ml)**	50	1.60 (80) +	0.32 (16) +	0.36 (18) +	2.28 (114) +
**0.10% (v/v) CMO + MMC (2.5*μ*g/ml)**	48	1.63 (78) +	0.38 (18) +	0.35 (17) +	2.35 (113) +
**0.50% (v/v) CMO + MMC (2.5*μ*g/ml)**	51	1.59 (81) +	0.27 (14) i	0.45 (23) +	2.31 (118) +
**1.00% (v/v) CMO + MMC (2.5*μ*g/ml)**	50	1.72 (86) +	0.48 (24) +	0.46 (23) +	2.66 (133) +

^1^The number of mutant spots is given in parenthesis. Symbols next to values signify the following: +, positive mutagenic effect;-, no mutagenic effect; i, inconclusive effect (p = 0.05); Statistical diagnosis according to Frei & Würgler [38].

The potential antigenotoxic activity of CMO was examined by co-treatment with the above concentrations of CMO and 2.5 *μ*g/ml MMC. As shown in Table 2, MMC, which was used as a positive control, evoked a statistically significant rise in all kinds of spots indicating genotoxic and recombinogenic activities and, thus, strengthening the validity of our system. When CMO was co-administered with MMC, no significant differences in any spot category were observed in the applied CMO concentrations (Table 2), indicating absence of antigenotoxic and antirecombinogenic activity.

In conclusion, our *in vivo* assay demonstrated that CMO lacks genotoxic or recombinogenic activity at the applied concentrations. Co-treatment with CMO and the genotoxic agent MMC revealed no statistically significant differences as compared to MMC alone.

## Discussion

In recent years there is an increasing international interest in mastic products derived from the plant *Pistacia lentiscus* (L.) var. chia (Duham) due to their antibacterial, antimicrobial, anti-inflammatory, antioxidant and anticancer activities [8–10]. Based on these data as well as on a recent work of our group [1] where we showed a protective role of CMW against the MMC-induced genotoxicity, in the present study we evaluated the cytotoxic and genotoxic effects of CMO, as part of establishing its safety profile, and estimated its antigenotoxic potential. For this purpose, the *in vitro* CBMN assay in cultured human lymphocytes and the *in vivo* Somatic Mutation And Recombination Test (SMART) in *Drosophila melanogaster* were employed as they allow detection of various genetic endpoints during the cell cycle or special developmental stages [14,15,43].

In both applied assays, CMO lacked genotoxic, mutagenic or recombinogenic effects, with the MN and wing spot frequencies not being statistically significantly different from the negative controls (Tables 1 and 2). However, statistical analysis of total spots in SMART test (Table 2) for the two highest concentrations (0.50, 1.00%) led to inconclusive results due to the increase in frequency of small spots, which in the case of 1.00% was found statistically significant. Even though in the latter concentrations the inconclusive results are interpreted as having minor biological significance, the positive result of small spots at the high concentration of 1.00% should not be overseen. Doi *et al*. [44] also indicated a promotion potential of Chios mastic gum on the formation of preneoplastic lesions in the rat liver at similar doses.

To our knowledge, there are no previous reports on the genotoxic potential of CMO. However, our results are in line with the absence of genotoxicity of another mastic extract, CMW, as well as of fruit extracts from *P*. *lentiscus* [1,45,46]. Moreover, one of the major constituents of CMO, β-myrcene, as well as some of its minor constituents [5,6,12] were found not to exert any genotoxic activity in a great number of *in vitro* and *in vivo* systems [47–57]. Regarding the other major constituent of CMO, α-pinene, although most studies have demonstrated lack of genotoxicity [47,50,54,56,57], it was once shown to compromise genomic stability [58]. CMO is a complex mixture of bioactive terpenes [5,6,12]; thus, the observed absence of genotoxicy could be attributed to synergistic and/or antagonistic actions among its constituents [56,59]. Furthermore, differences in results obtained using different concentrations and assays suggest that much attention should be given to better understand the underlying mechanisms and to determine the appropriate safety levels of mastic products as well as of all naturally-occurring agents.

The potential antigenotoxic activity of CMO was examined by co-treatment of human lymphocytes and *D*. *melanogaster* larvae with CMO and the mutagenic inducer MMC. MMC is an antibiotic that transforms into an alkylating agent and affects DNA synthesis, causes inter-strand cross-links in DNA and formation of DNA adducts [60–63]. It was found to be genotoxic in all *in vitro* and *in vivo* test systems in mammalian cells and animals and was clearly demonstrated as carcinogenic agent [1,64–71]. Accordingly, MMC was found to be genotoxic in both our *in vitro* and *in vivo* assays, inducing statistically significant increase in MN, BNMN and wing spots (Tables 1 and 2). The results obtained here by both assays did not demonstrate a protective effect of CMO against genotoxic action of MMC, indicating that it had no antigenotoxic activity in the specific concentrations and experimental conditions (Tables 1 and 2). Even though the two major compounds of CMO, i.e. α-pinene and β-myrcene, as well as a few others found in lower percentages (e.g. a-caryophyllene, limonene), have demonstrated some antigenotoxic potential [51,72–75], the fact that CMO could not afford any protection against MMC-induced genotoxic induction could be due to antagonistic effect among the CMO constituents or the different concentrations and assays applied in each study. However, other mastic products or extracts (e.g. CMW and *P*. *lentiscus* fruit extracts) have been shown to protect against genotoxic agents [1,45,46]. This difference could be due to the different composition of mastic products or extracts or to the CMO’s hydrophobic nature, which could interfere with the usual mechanisms underlying antimutagenic activity [73]. Indeed, CMO did not alter considerably the redox or detoxification mechanisms of different tissues [76].

When the cytotoxicity of CMO was evaluated, a significant decrease of CBPI values was observed at the highest concentrations of CMO (Table 1), as well as at all the concentrations of CMO and MMC mixtures, consistently with the previously reported cytotoxic/anticancer potential of CMO. Specifically, CMO inhibited Lewis Lung Carcinoma tumor growth both *in vitro* and *in vivo* [24] as well as the growth and survival of human K562 Leukemia Cells [23]. Moulos and colleagues [77] presented evidence concerning the molecular basis of CMO-induced inhibition of tumor cell growth and their gene ontology analysis revealed modifications on cell cycle/proliferation and survival among others. Furthermore, mastic gum extracts showed antitumor activity against human colorectal cancer [78]. Finally, the cytotoxic activity exhibited by CMO can be supported by literature data demonstrating that several of its constituents possess cytotoxic and anticancer potential [75,79–86]. However, most probably CMO’s cytotoxicity is the result of synergism, since combinations of phytochemicals had previously shown enhanced reactivity compared to individual compounds due to their additive and/or synergistic interactions [87]. Indeed, concerning CMO, its antibacterial activity was attributed to a cocktail of constituents including some of the trace elements [6].

In conclusion, our work provides evidence on the lack of genotoxic, mutagenic or recombinogenic activities of CMO under our *in vitro* and *in vivo* conditions. Although no antigenotoxic effect could be sustained, the absence of genotoxicity and the promising cytotoxicity is suggestive of a natural nontoxic product with pharmacological potential. The numerous and diverse properties of Chios mastic resin and its products warrant further research and an effort to identify specific constituents associated with different effects.
